# Surgical anatomy of the pectoralis major, pectoralis minor, latissimus dorsi and teres major for tendon transfer in irreparable subscapularis tendon tears

**DOI:** 10.1002/jeo2.70391

**Published:** 2025-08-05

**Authors:** Lin Lin, Qiang Liu, Fengyuan Zhao, Dingyu Wang, Hui Yan

**Affiliations:** ^1^ Institute of Sports Medicine Peking University Third Hospital Beijing China

**Keywords:** irreparable subscapularis tears, latissimus dorsi, pectoralis major, pectoralis minor, rotator cuff, tendon transfer, teres major

## Abstract

**Purpose:**

Tendon transfer of the pectoralis major (PM), pectoralis minor (Pm), latissimus dorsi (LD) and teras major (TM) is recommended for irreparable subscapularis (SSC) tears. This study aimed to describe the landmarks and neurovascular structures in the regions of the four muscles to facilitate their transfer to irreparable SSC tears.

**Study Design:**

Descriptive laboratory study.

**Methods:**

Twelve fresh‐frozen adult specimens were dissected and important neurovascular structures around the four tendons were identified. The relationships between the tendons and neurovascular structures were quantitatively investigated during tendon transfer procedures.

**Results:**

The average distance of medial pectoralis nerve (MPN) to the insertion of Pm on the coracoid was 6.5 cm (5.4–8.1 cm). The MPN travelled with the lateral thoracic artery (LTA) to pierce the PM with an average of 9.7 cm (8.3–12.1 cm) medial to the humeral insertion. Axillary nerve and posterior humeral circumflex vessels were above the superior margin of TM muscle with average of 0.8 cm (0.5–1.2 cm). These neurovascular structures crossed posterior to the plane of the LD and TM at 2.6 cm (1.9–3.3 cm) from the humeral insertion of these two muscles. The radial nerve (RN) and its motor branch to triceps were found to lie an average of 2.7 cm (2.0–4.3 cm) medial to the humerus at the superior border of the LD, and an average of 2.4 cm (1.8–3.5 cm) medial to the humerus at the inferior border of the TM. The neurovascular pedicles to the LD and TM were at an average of 12.7 cm (10.2–15.6 cm) and 7.0 cm (5.6–8.5 cm) to the humeral insertions, respectively.

**Conclusions:**

Our results clarify the complex local anatomic structures of the PM, Pm, LD and TM for tendon transfer to treat irreparable SSC tears and provide potentially useful references for tendon transfer.

**Level of Evidence:**

Not applicable.

**Abbreviations**
AB: aponeurotic band
LD: latissimus dorsi
LHT: long head of the triceps
LPN: lateral pectoral nerve
LTA: lateral thoracic artery
MCN: musculocutaneous nerve
MPN: medial pectoralis nerve
PM: pectoralis major
PM: pectoralis major
RN: radial nerve
SSC: subscapularis
TM: teres major

## INTRODUCTION

Large subscapularis (SSC) tears lead to horizontal imbalance of the shoulder accompanied with pain and weakness during active internal rotation [15, 33]. Irreparable SSC tears, typically characterised by marked tendon retraction, poor tendon quality and severe fatty muscle infiltration, are associated with significant shoulder dysfunction and morbidity [10, 36, 37].

Several reconstruction procedures exist to reduce pain, recover internal rotation and stabilise the humeral head anterosuperior escape. Tendon transfer is recommended to reconstruct SSC function in patients with irreparable SSC tears, especially in young and active patients. Pectoralis major (PM) [1, 12, 14], pectoralis minor (Pm), latissimus dorsi (LD) [11, 18] and teres major (TM) [3] tendon transfers were performed in patients with irreparable anterosuperior cuff tears with improved clinical outcomes.

Traditionally, open procedures were performed to harvest and release the tendons during surgery [11, 12, 28]. With the advancement of arthroscopic techniques, procedures for all‐arthroscopic and arthroscopic‐assisted dissection and the release and fixation of the PM, Pm and LD/TM have recently been developed [16, 18, 24, 38]. Understanding the regional anatomy is crucial to avoid potential neurovascular injury because arthroscopic tendon transfer surgery is technically demanding. There is a paucity of information on the anatomy of the LD, TM, MT and LT muscles regarding anterior transfer to restore SSC function. This study aimed to describe the essential landmarks and neurovascular structures in the PM, Pm, LD and TM muscles to facilitate their transfer to irreparable SSC tears.

## MATERIALS AND METHODS

Ethical approval for this study was obtained from Peking University Third Hospital (IRB00006761‐M2024419). Twelve freshly frozen adult forequarter specimens were dissected. No evidence of shoulder surgery or anatomical deformities was found in any of the specimens. Among the cadavers, there were seven male and five female subjects. The mean age was 53 years (range, 45–64 years); six right and six left shoulders were used.

Dissection was performed using the extended anterior deltopectoral approach. The conjoint tendon and coracoid tip were identified, and the lower border of the SSC muscle and upper border of the PM tendon were exposed. The insertions of the PM, Pm, LD and TM tendons were identified, and their insertional widths and lengths were measured. After the detachment of the PM tendon, the Pm tendon and muscles were exposed. The lateral pectoral nerve (LPN) and medial pectoral nerves (MPN) were inserted into the PM and Pm. The distances from the LPN and MPN to the tendinous insertions of the PM and Pm, respectively, were recorded. The distances between the neurovascular structures (axillary nerve, posterior humeral circumflex vessels, radial nerve [RN] and motor branch to the long head of the triceps [LHT]) and the insertion at the superior and inferior margins of the LD and TM tendons were measured. The thoracodorsal and subscapular nerves were inserted into the LD and TM muscles, respectively. The distances from the tendinous insertions of the LD to the thoracodorsal nerve pedicles and TM to the subscapular nerve pedicles were measured. Measurements were performed using digital calipers by a single surgeon, with the arm at the side in neutral rotation and gentle tension applied to the muscles. The accuracy of the calipers was 1 mm. The distances were measured once.

## RESULTS

### Tendon dimensions

**Figure 1:**
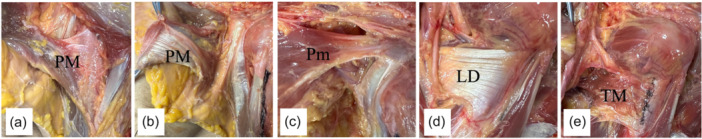
Tendon insertions of pectoralis major (PM), pectoralis minor (Pm), latissimus dorsi (LD), and teres major (TM). (a) Anterior view of PM. (b) Posterior view of PM. (c) Pm. (d) Latissimus dorsi. (e) Teres major.

The Pm insertion was targeted at the medial side of the coracoid in all specimens. The PM is a triangular muscle located anterior to the SSC and inferior to the deltoid muscle. The tendon was successfully split into the anterior and posterior laminae in each specimen. The tendon of the anterior lamina originated from the clavicular muscular portion, and the posterior lamina originated from the inferior sternal portion. The superior margin of the PM was anterior to that of the LD. The LD tendon was always inserted anterior to the TM tendon in the humerus. The LD was inserted into the proximal humerus with a distinct tendon (eight cases) or a conjoined tendon with the TM along the inferior border (four cases). The LD and TM muscles were adhered to their dorsomedial edges approximately 3.0 cm proximal to their insertion points. The average widths and lengths of the PM, Pm, LD and TM tendons are shown in Table 1. Typical images of the PM, Pm, LD and TM tendons are shown in Figure 1.

**Table 1:** The tendon dimensions of PM, pectoralis minor (Pm), latissimus dorsi and teres major.

	Width at insertion (cm)	Length of tendon (cm)
PM	6.1 (5.2–6.8)	*N* a
Pm	2.1 (1.6–2.5)	3.1 (2.8–3.9)
Latissimus dorsi	3.6 (2.8–4.7)	8.2 (7.1–11.2)
Teres major	4.0 (3.6–5.3)	3.5 (2.5–4.0)

a The length of the anterior laminae of the pectoralis major (PM) tendon was 3.0–4.9 cm at the upper border and gradually narrowed from the proximal to distal ends. The length of the posterior lamina of the PM tendon was 6.8–11.7 cm.

### Neurovascular structures adjacent to PM and Pm

The brachial plexus was posterior to the Pm muscle and encased in fat tissue. The LPN was found to enter the clavicular head at a mean distance of 12.0 cm (range, 9.2–14.1 cm), positioned medial to the humeral insertion point of PM. The LPN passes medially to the Pm and enters the clavicular head superior to the intermuscular septum.

**Figure 2:**
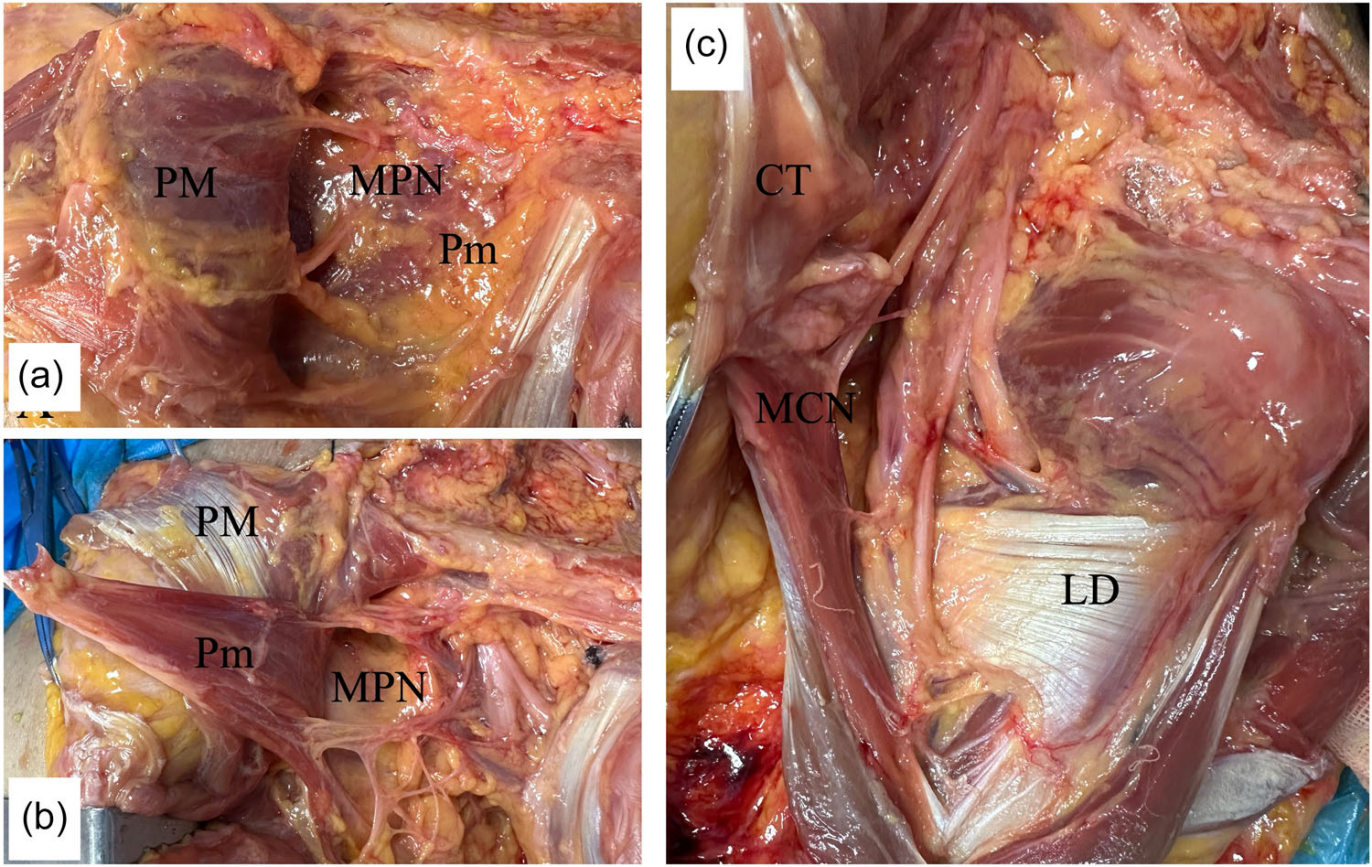
(a) The medial pectoralis nerve (MPN) passing through the pectoralis minor (Pm) muscle as two branches to the pectoralis major (PM) muscle. (b) Two branches of the MPN passing through the Pm. (c) The musculocutaneous nerve (MCN) at the undersurface of the coracobrachialis. CT, conjoint tendon; LD, latissimus dorsi.

The MPN passes through the Pm before entering its undersurface. The inferior fibres of the PM were innervated by the MPN, which was accompanied by the lateral thoracic artery (LTA). The MPN pierced the PM at an average distance of 10.7 cm (8.3–13.9 cm), medial to the humeral insertion, and 2 cm proximal to the inferior edge. The MPN passed through the PM muscle as a single branch in five specimens and passed through the Pm muscle as two branches in seven specimens. The average distance of MPN to the insertion point of Pm on the coracoid was 6.5 cm (5.4–8.1 cm) (Figure 2).

The musculocutaneous nerve (MCN) entered the undersurface of the coracobrachialis at a distance of 5.4–6.1 cm distal from the coracoid origin. The mean vertical distance of MCN to the tip of coracoid was 2.2 cm (1.5–2.8 cm) (Figure 2).

### Neurovascular structures adjacent to LD and TM

#### Axillary nerve, anterior and posterior humeral circumflex vessels

**Figure 3:**
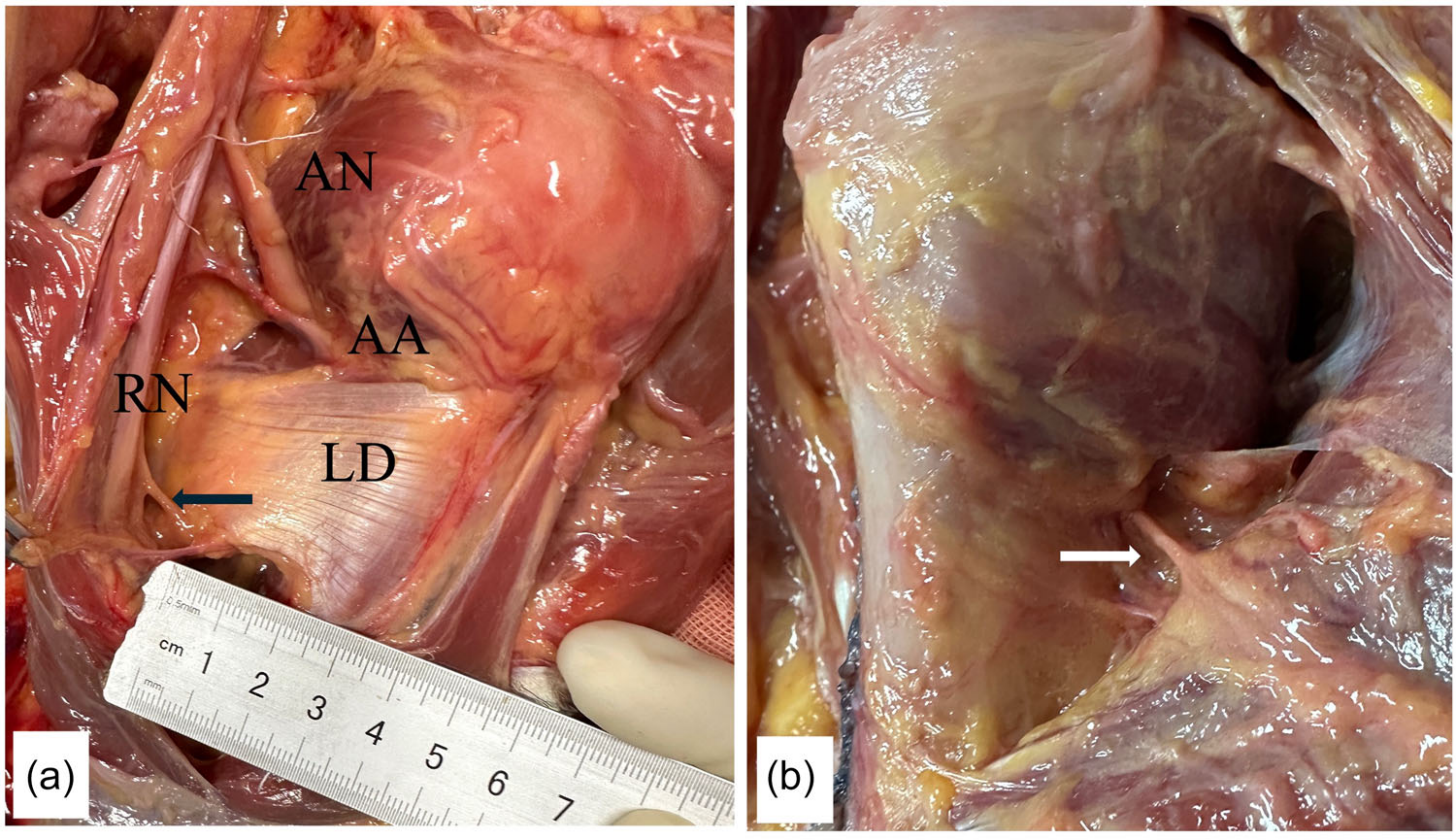
Neurovascular structures adjacent to latissimus dorsi (LD) and teres major (TM). (a) Anterior view. Anterior humeral circumflex vessels act as the land markers of the superior margin of humerus insertion of LD tendon. The radial nerve (RN) and the motor branch to the long head of the triceps are wrapped in fatty tissue and cross the LD and TM tendon anteriors. Black arrow: the motor branch of RN to the long head of the triceps. AA, axillary artery; AN, axillary nerve. (b) Posterior view. White arrow: the posterior branch of the axillary nerve exits the quadrilateral space.

The anterior humeral circumflex vessels were the land markers of the superior margin for the humeral insertion of the LD tendon (Figure 3). The axillary nerve and posterior humeral circumflex vessel were just above the superior margin of LD and TM muscle at an average distance of 0.8 cm (0.5–1.2 cm) (Figure 3). These neurovascular structures cross posteriorly to the plane of the LD and TM at a distance of 2.6 cm (1.9–3.3 cm) from the humeral insertion points of these two muscles.

#### The radial nerve (RN) and the motor branch to the LHT

The RN and motor branches of the LHT were encased in fatty tissue and passed through the LD and TM tendon anteriors. The motor branch of the LHT was immediately anterior to that of the LD (Figure 3). With the arm at the side in neutral rotation, the distance of RN to the humerus at the superior border of the LD and inferior border of the TM was 2.7 cm (2.0–4.3 cm) and 2.2 cm (1.8–3.5 cm), respectively. The RN and motor branches were medial to the conjoint tendon muscle.

#### Aponeurotic band between the triceps and the LD

**Figure 4:**
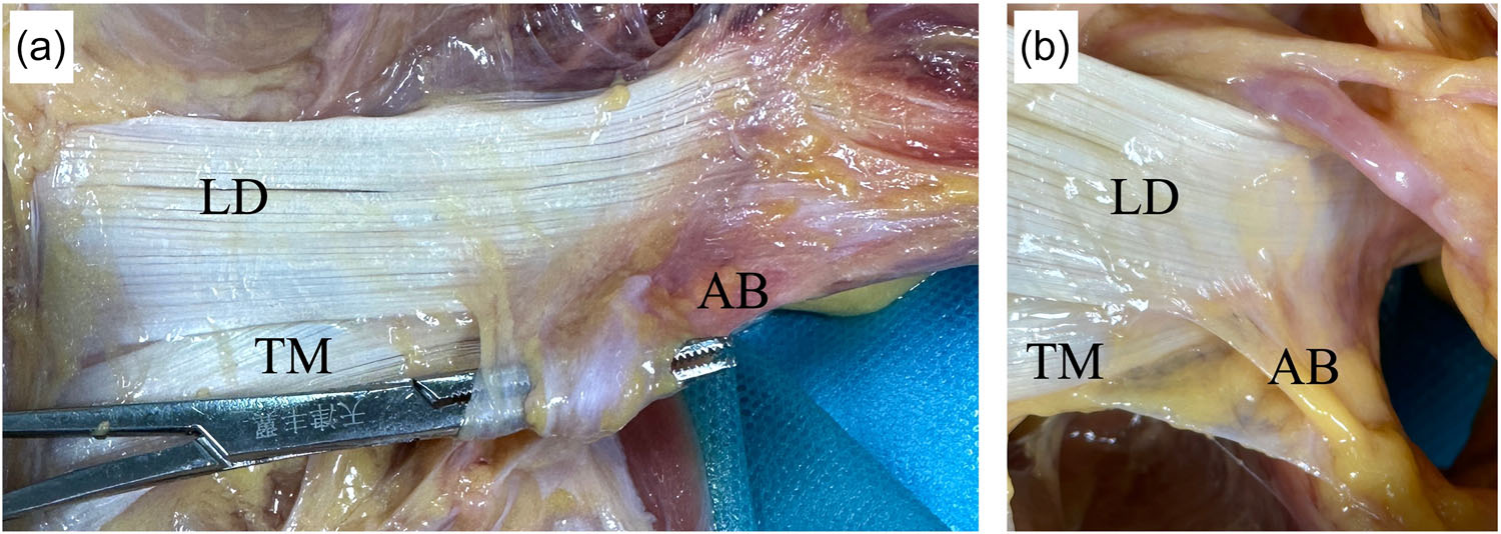
Aponeurotic band (AB) between the triceps and the latissimus dorsi (LD). TM, teres major. (a) The anterior view. (b) The side view.

There was an aponeurotic band medial to the RN between the triceps and the LD with mean distance 4.1 cm (3.7–5.6 cm) between aponeurotic band and RN (Figure 4) in each specimen.

#### Neurovascular pedicles of the LD and TM

**Figure 5:**
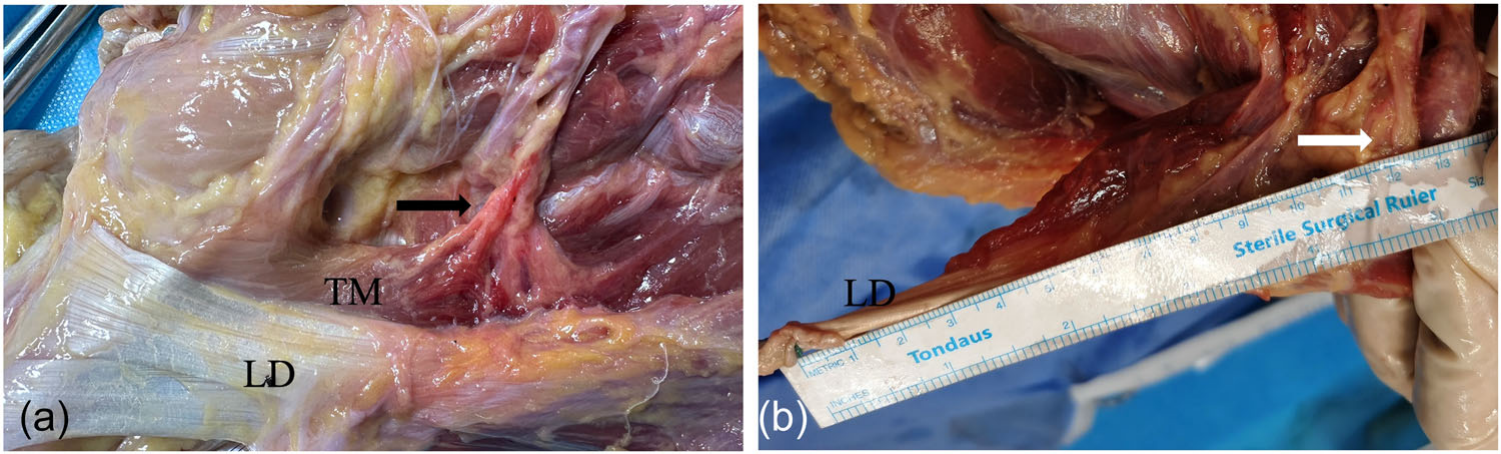
Neurovascular pedicles of the latissimus dorsi (LD) and teras major (TM). (a) The lower subscapular neurovascular pedicle entersed the anterior muscle belly of the TM. Black arrow: the lower subscapular neurovascular pedicle. (b) The thoracodorsal neurovascular pedicle is inserted on the anterior muscle belly of the LD. White arrow: the thoracodorsal neurovascular pedicle.

The thoracodorsal neurovascular pedicle entered the anterior muscle belly of the LD at 12.7 cm (10.2–15.6 cm) medial to its humeral insertion point (Figure 5). There were two neurovascular branches to the muscle belly of the LD in six shoulders and one terminal neurovascular branch with a broad insertion in six shoulders. The lower subscapular neurovascular pedicle entered the anterior muscle belly of the TM at 7.0 cm (5.6–8.5 cm) medial distance to its humeral insertion point (Figure 5).

## DISCUSSION

Our results comprehensively show the anatomic relationships of the PM, Pm, LT, and TM and the essential neurovascular structures in terms of tendon transfer for irreparable SSC tears. Anatomical structures are at risk during the release of these tendons and muscles. The distance from the neurovascular structures to the tendons was measured and recorded.

PM transfer is considered the gold standard and is widely performed [1, 12, 14]. The PM anatomy has been widely investigated for the repair of PM tendon tears and tendon transfer in SSC [6, 13, 17, 26]. Traditionally, it was believed that the tendon rotated on itself, with the fibres of the most inferior part of the sternal head inserted superiorly and the more superior fibres inserted inferiorly [2, 31]. However, a dissection and comprehensive digital reconstruction study showed, ‘No twisting of either the PM muscle or tendon was noted’ [13]. The two to three inferior segments are relatively smaller than the superior segments, and the anterior axillary fold may persist when they are only partially torn, complicating the clinical diagnosis [13]. Transfer of the entire PM tendon demonstrated good clinical results in cases with long‐term follow‐ups of more than 20 years [12]. The anterior or posterior layer of the PM has also been studied in the literature using conjoint tendon methods [17, 33, 37]. In the present study, it might have been difficult to split the posterior layer of the tendon, and the transfer of the entire bulky PM under the conjoint tendon might have caused MCN neurapraxia. MPN were found to be at risk, whereas LPN were not at risk in the split technique.

The Pm tendon has also been used to treat irreparable SSC tears in patients with lesions in the upper portion of the SSC tendon [28]. The clinical anatomy of Pm has also been investigated by several authors [7, 23, 32, 34]. The anatomic relationship between the costal insertions of the PM and Pm muscles is highly variable [34]. In 1897, LeDouble and colleagues [7] classified insertional variants into three types: type I variants were the most common, type II variants were uncommon, and type III variants were rare. However, a recent study showed that type II variants are less uncommon than originally believed [22]. Klepps et al. [19] reported that the MPN sometimes passes laterally to the Pm. Jennings et al. [17] confirmed that the MPN passed lateral to the Pm in 4 out of 24 cadavers. The MPN travels with the LTA and is at risk during separation of the two muscular units. These variations in tendon insertion and MPN paths should be noted when performing open and arthroscopic Pm tendon transfer [24].

Recently, LD tendon transfer has gained attention as a potential treatment for patients with irreparable SSC tears [11, 18]. A biomechanical study showed that anterior LD tendon transfer restored the anteroposterior force couple, prevented anterosuperior humeral head shifts, and improved glenohumeral kinematics [5]. Combined LD and TM tendon transfer in patients with irreparable anterosuperior cuff tears improves clinical and radiological outcomes with low complication rates [3].

The LD has been suggested as a more suitable musculotendinous donor than the PM and Pm because of its posterior origin from the chest wall and ability to mimic the line of pull of the SSC [9, 20]. Several studies have investigated the clinical outcomes of anterior LD transfer during short‐term follow‐ups [11, 18, 25, 35]. A lower trapezius transfer and a teres major transfer were found to be feasible in a cadaveric study with low risk of nerve compression [8, 21]. A minor rhomboid transfer was proposed; however, it was advised against the risk of tendon luxation during external rotation [8]. To the best of our knowledge, no clinical studies have reported these transfers. The anatomies of the LD and TM in our study were consistent with those in previous studies [29, 30]. The LD and TM tendons have been successfully used to treat posterosuperior irreparable rotator cuff tears. For the anterior transfer of the LD and TM tendons, local neurovascular structures are at risk during the release and detachment of these tendons. Care must be taken to avoid damage to neurovascular structures during LD/TM release. To improve excursion during the transfer of the LD and TM, fascial connections between the muscle bellies of the TM and LD should be released. The average tendon length was 3.5 cm for the TM and 8.2 cm for the LD. Although the excursion of these combined tendons was lower than that of the LT alone, transfer of the conjoined LD/TM tendon could be performed successfully.

This study had some limitations. (1) The inherent potential for changes in soft tissue relationships during measurements depends on the cadaveric dissections. (2) Shoulder dissection and measurements were performed on a relatively small number of specimens. Cadaveric specimens are valuable; typically, fewer than 10 specimens have been used in previous biomechanical studies [4, 27]. (3) Forequarter samples were used for dissection. Therefore, comments on the amount of musculotendinous unit mobilisation required for transfer are based on both previous clinical experience and cadaveric investigations.

This cadaveric study revisited and further clarified the complex local anatomical structures of the PM, Pm, LD and TM for tendon transfer in the treatment of irreparable SSC tears. The strength of this study was that it confirmed the previously published anatomic descriptive data while providing potentially useful references for tendon transfer especially for arthroscopic techniques.

## AUTHOR CONTRIBUTIONS

All authors contributed to the study conception and design. Material preparation, data collection and analysis were performed by Lin Lin, Hui Yan, Qiang Liu and Fengyuan Zhao. The first draft of the manuscript was written by Lin Lin and all authors commented on previous versions of the manuscript. All authors read and approved the final manuscript.

## CONFLICT OF INTEREST STATEMENT

The authors declare no conflicts of interest.

## ETHICS STATEMENT

Ethical approval for this study was obtained from Peking University Third Hospital (IRB00006761‐M2024419). The ethics committee waived the written informed consent from the donor for this research, as people sign the consent form for organ donation, they have already agreed to the use of the donated organs for scientific research and anatomical teaching purposes.

## Data Availability

The data that support the findings of this study are available from the corresponding author upon reasonable request.
